# Hierarchical Conformational Analysis of Native Lysozyme Based on Sub-Millisecond Molecular Dynamics Simulations

**DOI:** 10.1371/journal.pone.0129846

**Published:** 2015-06-09

**Authors:** Kai Wang, Shiyang Long, Pu Tian

**Affiliations:** 1 School of Life Sciences, Jilin University, Changchun, China; 2 MOE Key Laboratory of Molecular Enzymology and Engineering, Jilin University, Changchun, China; Hong Kong University of Science and Technology, HONG KONG

## Abstract

Hierarchical organization of free energy landscape (FEL) for native globular proteins has been widely accepted by the biophysics community. However, FEL of native proteins is usually projected onto one or a few dimensions. Here we generated collectively 0.2 milli-second molecular dynamics simulation trajectories in explicit solvent for hen egg white lysozyme (HEWL), and carried out detailed conformational analysis based on backbone torsional degrees of freedom (DOF). Our results demonstrated that at micro-second and coarser temporal resolutions, FEL of HEWL exhibits hub-like topology with crystal structures occupying the dominant structural ensemble that serves as the hub of conformational transitions. However, at 100*ns* and finer temporal resolutions, conformational substates of HEWL exhibit network-like topology, crystal structures are associated with kinetic traps that are important but not dominant ensembles. Backbone torsional state transitions on time scales ranging from nanoseconds to beyond microseconds were found to be associated with various types of molecular interactions. Even at nanoseconds temporal resolution, the number of conformational substates that are of statistical significance is quite limited. These observations suggest that detailed analysis of conformational substates at multiple temporal resolutions is both important and feasible. Transition state ensembles among various conformational substates at microsecond temporal resolution were observed to be considerably disordered. Life times of these transition state ensembles are found to be nearly independent of the time scales of the participating torsional DOFs.

## Introduction

During last two decades, great progress has been made in the study of major conformational transitions for some functionally important proteins [1–10]. Such transitions usually exhibit spatial displacement of some structural elements up to a few nanometers, often involve collective motions of many residues and occur on time scales ranging from microseconds to milliseconds and beyond. Computationally, elastic network model (ENM) based methodologies have been successfully applied to explain functional relevance of such large scale conformational change [11, 12]. Unfortunately, the complexity of protein conformational space does not stop here. For a given protein, each major conformation is compatible with many conformational substates (CSs) that have different combinations of side chain and backbone torsional states. Transitions among these CSs manifest themselves as rotation of backbone dihedrals *ϕ*, *ψ* and side chains on time scales ranging from sub-nanoseconds to micro-seconds and beyond. These motions are qualitatively termed as flexibility, which is widely acknowledged to be important or sometimes critical in various molecular interactions [13]. However, due to the astronomically large number of possible CS, not much effort has been invested in studying CS transitions, distributions and organization in conformational space. A series of seminal experimental studies on the rebinding dynamics of CO by myoglobin provided strong support for hierarchical organization of free energy landscape (FEL) in native proteins [14, 15]. However, multi-dimensional native FEL of protein is only projected onto one measurable dimension in these experiments, the topological organization of CS at various temporal resolutions and corresponding detailed structural information of the whole protein is not available.

Native (functional) protein FEL has been analyzed in detail by utilizing atomistic molecular dynamics (MD) simulation trajectories that are a few hundred nanoseconds to microseconds-long [16]. Major methodologies are various forms of principal component analysis (PCA) that are based upon either cartesian or internal coordinates (mainly dihedral angles). Hierarchical organization of CS is supported by these studies on the FEL of various proteins in the vicinity of starting crystal structures. However, in addition to limitation of low dimensions, time scale is quite limited and conclusions made may not be readily extended to the global organization of native FEL. The majority of experimental and computational studies of FEL for native proteins up to date are designed to explain how given known functions/properties are related to the underlying FEL. Reduction of dimension (such as projection of FEL onto certain experimentally measurable quantities [14] or various forms of PCA [16]) is an effective means. With increasing environmental challenges (e.g. nanomaterials and new drugs) faced by biological systems, our concern is expanded from interactions among endogenous biological molecular partners to interactions between interested biomolecules and large number of chemical entities. Therefore, to transform the role of conformational analysis of proteins from explaining known to predicting a wide spectrum of molecular interactions, it might potentially be helpful to study global native FEL of proteins in as high dimensional space that we may understand as possible. Despite great progresses that have been achieved by the docking community [17, 18], it is well acknowledged that obtaining accurate structures of complexes from structures of their comprising proteins is a difficult task. A sufficiently complete backbone native structural ensemble was demonstrated to improve docking significantly [19]. Proper treatment of CS (or flexibility) has become a major bottleneck in improving prediction power of docking (both protein-ligand and protein-protein) methodologies [13, 20]. Therefore, a deeper fundamental understanding of the distribution and organization of CS is highly desired.

Great breakthroughs have been achieved in understanding folding mechanisms of small proteins by millisecond MD simulations [9, 21–24]. Despite acknowledged uncertainties [24–27], many MD simulation folding studies [24, 28] demonstrated that modern molecular mechanical force fields are reasonably accurate to distinguish the native ensemble from many unfolded ensembles. Inspired by successful folding studies with MD simulations [24], we posed the following questions: i) how are CS organized within conformational space of native proteins at various temporal resolutions (or free energy hierarchies); ii)how does FEL of native proteins generated by molecular mechanical force fields compare with available experimental data and; iii) among astronomically large numbers of possible CS, what fractions are of realistic significance in determining physiochemical properties and biological functions of a given protein. These questions are difficult to tackle in a general sense. We chose to use HEWL as a model protein and CHARMM22 (with CMAP) as a typical set of force field parameters. This particular case is likely to serve as a representative for many other globular proteins. We generated collectively 0.2*ms* MD trajectories for HEWL in explicit solvents (water and NaCl). Based on distributions of backbone dihedrals, 50,000,000 snapshots were clustered at temporal resolutions ranging from ∼ *ns* to ∼ 20*μs*. It is found that at *μs* and coarser temporal resolutions, native FEL exhibit a hub-like topology with the dominating ensemble, which harbors nearly all crystal structures, serving as the hub connecting smaller structural ensembles among which mutual transitions happen occasionally. At 100*ns* and finer temporal resolutions, however, HEWL clusters form network-like organization, and crystal structures are associated with significant ensembles that form kinetic traps. The number of backbone CS that is of statistical significance is found to be rather limited even at *ns* temporal resolution, revealing strong conformational correlations among backbone dihedrals. Comparison with available crystal structures indicates that backbone torsional state transitions involved in various molecular interactions span time scales ranging from *ns* to ∼ 10*μs*.

## Results

### Sets of HEWL CS ensembles at multiple temporal resolutions

**Fig 1:**
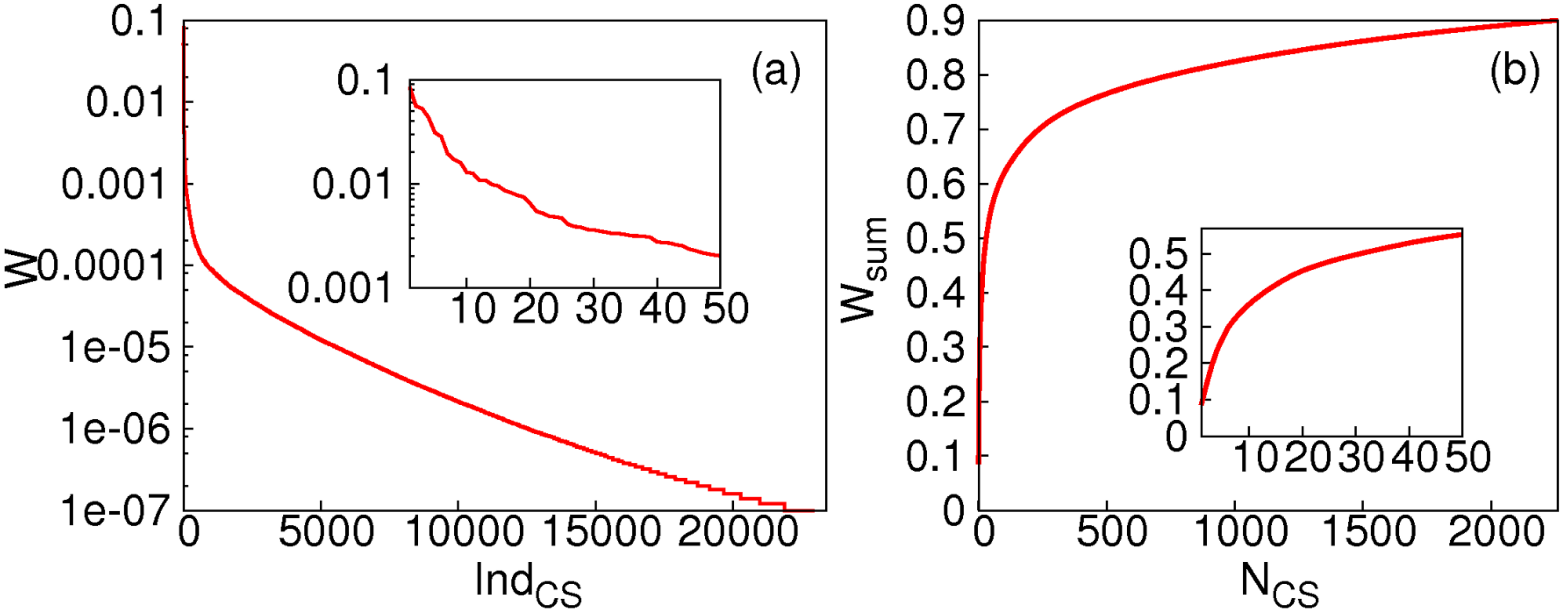
a) Statistical weight (*W*) of the largest 23400 (out of 40356) CSs at time resolution *T*_4_, the horizontal axis are indices for CS (*Ind*_*CS*_). Inset: *W* for the 50 largest CSs. b)Collective statistical weight (*W*_*sum*_) of the N largest CSs (*N*_*CS*_) at time resolution *T*_4_. Inset: magnification for small *N*_*CS*_.

To explore the native conformational space of HEWL, we generated 2000 100 − *ns* MD trajectories and saved 50,000,000 structures (with 4*ps* intervals). To verify that our MD trajectories have achieved sufficient sampling for the native HEWL conformational space, we performed clustering on various subsets of trajectories. The high level agreement regarding the relative importance of significant clusters indicate that our sampling of the HEWL conformational space is reasonably sufficient (Fig. A in S1 File). Distributions were plotted for each *ϕ* and *ψ* that exhibit multiple-peaks (Table A and Fig. B in S1 File) under given temporal resolutions (Table 1), and were utilized to assign all the saved structures to different clusters and transition state ensembles. Briefly, 127 backbone dihedrals with two-major-peak distributions were defined as two-state torsional DOFs, and were divided into 5 different temporal resolutions according to the number of observed transitions between the two torsional state of each selected torsion. Under a given time resolution (say *T*_2_), two snapshots belong to the same cluster if and only if for each of the torsional DOF that has the same or coarser time resolutions (i.e. two-state torsional DOFs at *T*_0_, *T*_1_ and *T*_2_), they share the same torsional state (see Methods for details). The number of clusters obtained is listed for five specified temporal resolutions (*T*_0_, *T*_1_, *T*_2_, *T*_3_ and *T*_4_, see Table 1). At the temporal resolution *T*_4_(∼ *ns*), the statistical weight (*W*) of snapshots in each cluster (used interchangeably with CS hereafter) is plotted in Fig 1a and the percentage of snapshots in the largest *n* clusters (*W*_*sum*_) were plotted as a function of *n* in Fig 1b (See Fig. C in S1 File for similar plots at other temporal resolutions). Apparently, the number of CS observed in our simulations is quite limited, and the number of CS that have statistical significance is much less (with only 868 CSs has a weight larger than 0.01% at the temporal resolution *T*_4_). These observations suggest that quantitative analysis of significant CS at temporal resolutions as fine as *ns* is potentially feasible, at least for backbone conformational space. It is noted that the number of clusters depends highly on the temporal resolution used to perform clustering, and is much smaller for coarser temporal resolutions (Table 1). Statistics at temporal resolution *T*_0_ is quite limited, and our analysis and discussion hereafter focus on temporal resolutions *T*_1_ through *T*_4_.

**Table 1:** Five temporal resolutions utilized for hierarchical backbone conformational analysis of HEWL. Corresponding number of conformational substates is provided in the right column.

Temporal resolution	*N*_*Trans*_	200(*μs*)/*N*_*Trans*_	*N*_*CS*_
*T*_0_	0< *N*_*Trans*_ < = 10	20*μs* to 200*μs*	10
*T*_1_	10< *N*_*Trans*_ < = 100	2*μs* to 20*μs*	35
*T*_2_	100< *N*_*Trans*_ < = 1000	200*ns* to 2*μs*	434
*T*_3_	1000< *N*_*Trans*_ < = 10000	20*ns* to 200*ns*	12967
*T*_4_	10000< *N*_*Trans*_ < = 100000	2*ns* to 20*ns*	40356

**Fig 2:**
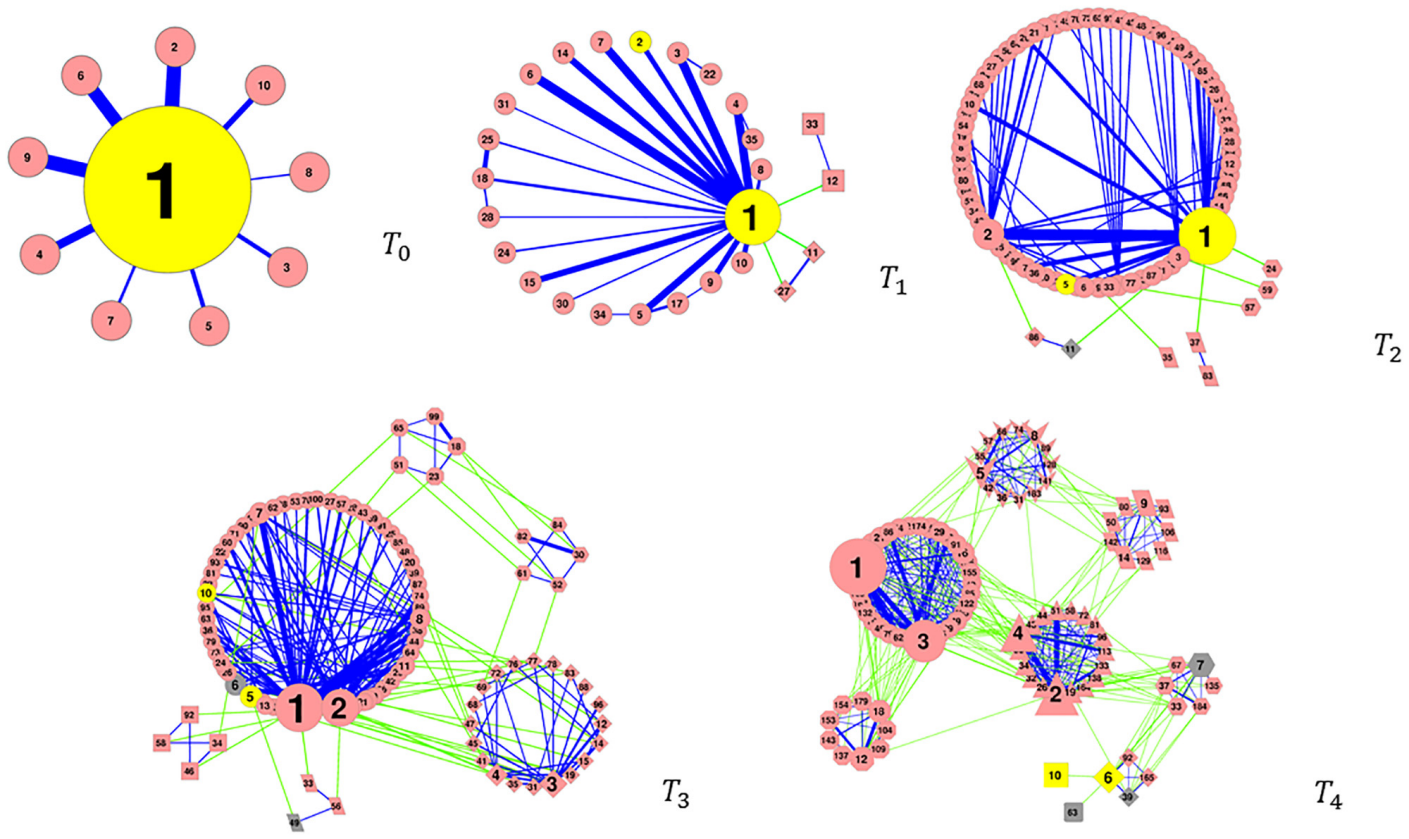
CS organization at temporal resolutions *T*_0_, *T*_1_, *T*_2_, *T*_3_ and *T*_4_. Each line (edge) between the two defining nodes (representing CSs) indicates occurrence of direct transitions between these two CSs. Sizes of nodes are indicative of CS’s statistical weight and thickness of lines are indicative of the number of direct transitions (but not strictly to the proportion). Blue lines represent transitions betweens clusters that merge to the same cluster on the preceding coarser temporal resolution, while green lines represent transitions between clusters that belong to different clusters on the preceding coarser time resolution. Yellow nodes harbor HEWL crystal structures in both single and complex forms, gray nodes harbor HEWL crystal structures in complexes with other proteins, and the remaining nodes in pink represent CSs that do not harbor any crystal structures. Only significant CSs are shown.

The hierarchical organization of obtained HEWL CS in backbone conformational space is plotted in Fig 2(a-d). At temporal resolution *T*_1_, a hub-like topology is observed and the dominating ensemble serves as the hub of conformational transitions. At finer temporal resolutions (*T*_3_ and *T*_4_), CSs of HEWL exhibit network-like organization, and both the average number of neighboring clusters (that have direct transitions to and from a given cluster) and its variation increases (Fig 2). In Fig 2, Approximately half of the lines represent more than a dozen direct transitions between clusters, and a significant fraction (20% to 30% dependent upon temporal resolutions) of lines represent hundreds or more inter-cluster transitions. This fact further suggest that our sampling is reasonably sufficient.

### Comparison with wild type crystal structure ensemble

**Fig 3:**
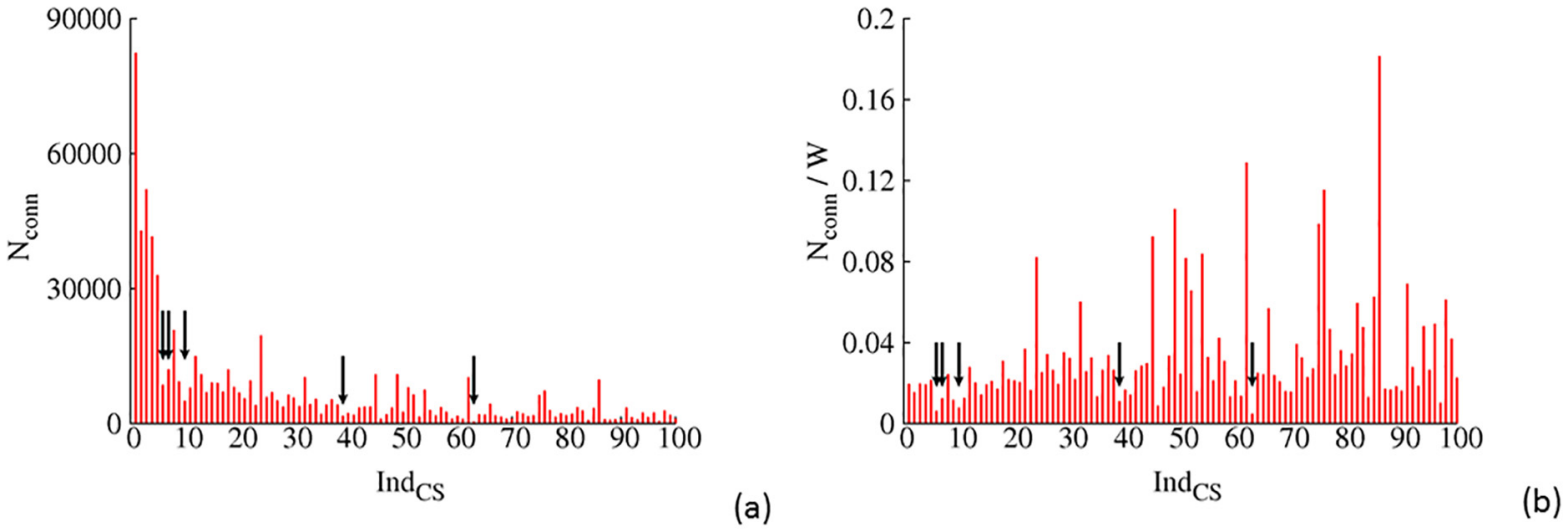
Extent of inter-cluster transitions for the largest 100 CSs at temporal resolution *T*_4_, CSs that harboring crystal structures are indicated by arrows. a) The total number of transitions (*N*_*conn*_), b)upon normalization with the corresponding statistical weight (*N*_*conn*_/*W*).

To examine the experimental relevance of our obtained CSs, 120 crystal structures of wild type HEWL are clustered using the same criteria established for MD snapshots. As shown in Fig 2 and Table B in S1 File, At temporal resolutions *T*_1_ and *T*_2_, nearly all examined crystal structures are located in the dominating structural ensemble from our simulations. However, at *T*_3_ and finer temporal resolutions (≤ 200*ns*), crystal structures are scattered in more and more clusters that are significant but not dominant anymore. This observation might indicate the deficiency of the utilized force fields in differentiating CS on such fine temporal resolutions. Dominance by a structural ensemble that does not correspond to crystal structure(s) has been attributed to inaccuracy of force fields [9]. Nevertheless, we need to note that the simulation condition is quite different from crystallization conditions of any utilized crystal structures. Finally a given crystallization process is not guaranteed to capture the most populous solution conformational substate at arbitrarily given temporal resolutions. Therefore, the exact reason for mismatch between dominant simulated ensembles and crystal structural ensemble (CSE) at fine temporal resolutions is not clear. It is likely that all three factors contribute simultaneously. As shown in Fig 3a, At temporal resolution T4, clusters harboring CSE are observed to have significantly less inter-cluster connections when compared with other clusters that have similar statistical weight (i.e. neighbors on the X axis, inter-cluster connections normalized by statistical weight are shown in Fig 3b). It is noted that such “kinetic trap” property is only observed on relatively fine temporal resolutions (*T*_3_ and *T*_4_). At coarse temporal resolutions (*T*_1_ and *T*_2_), each major CSE seems to be “contaminated” by many non-CSE solution states on finer temporal resolutions (Fig. D in S1 File).

**Fig 4:**
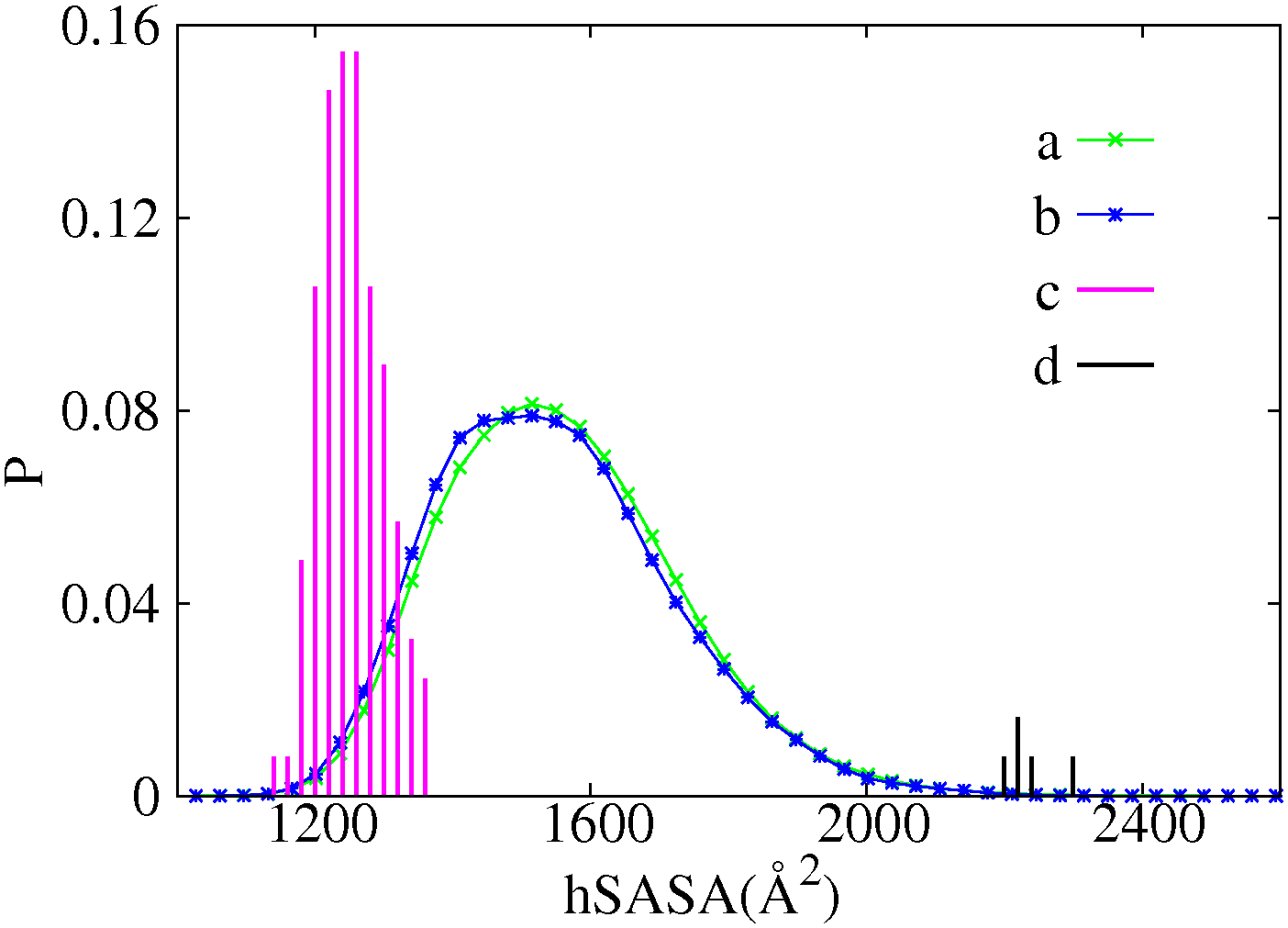
Probability distribution of *hSASA* for the crystal structure ensemble and MD simulation ensembles. Green line a): from trajectories that originate from crystal structures without sugar substrates/inhibitor bound; Blue line b): from trajectories that originate from crystal structures with sugar substrates/inhibitor bound; Purple impulses c): from crystal structure ensemble without sugar substrates/inhibitor bound; Black impulses d): from crystal structures with sugar substrates/inhibitor bound.

We analyzed distributions of crystal structures among the clusters established from MD trajectories at time resolutions *T*_1_ through *T*_4_ (see supporting text for details). Briefly, for backbone torsional transitions associated with various molecular interactions that were captured by crystal structures, their corresponding time scales were found to vary from ∼ *ns* to multiple *μs*. Functionally relevant substrate-bound and inhibitor-bound structures, while sharing large hydrophobic solvent accessible surface area (hSASA) (Fig 4), locate in different clusters at temporal resolutions *T*_3_ and *T*_4_. Site for change of backbone torsional states associated with molecular interactions do not necessarily co-localize with corresponding interaction residues. This observation is in agreement with the concept that all dynamic proteins are potentially allosteric [29]. All HEWL crystal structures that are in complexes with antibodies and a few other small proteins locate in significant clusters obtained from our simulations of free form HEWL. Therefore, the conformational selection mechanism [30] seems to dominate for interactions of HEWL and its protein partners.

### Comparison with mutant crystal structure ensemble

There are many crystal structures available in PDB for a wide variety of HEWL mutants (see Table C in S1 File). We were interested in examining how point mutations impact selection of potential CSE by crystallization. To this end, we performed the same clustering procedure with 29 mutant structures and listed the results in Table C in S1 File. It is found that mutants crystal structures are located in the same clusters as the wild type ones under similar crystallization conditions. As shown in Fig. E in S1 File, analyzed mutations are distributed across the whole protein. Therefore, at least for this set of mutants, single (and multiple) point mutations do not significantly influence the net result of CSE selection during crystallization. This observation suggests that for HEWL, crystallization conditions are more important than point mutations in selecting CSE. Therefore, caution has to be applied in explaining any structural change observed in mutant crystal structures under different crystallization conditions. In crystallization attempts, adjusting solution condition is a more widely utilized strategy than point mutations. Such practice is consistent with our current observation. The rational might be that solution conditions directly impact many surface residues that are more relevant in crystal contacts, and consequently has a larger probability to result in significant change of interaction networks that determine selection of CSE in target protein molecules. This observation is likely to be true for other globular proteins.

### Physical properties of major CS and transition state ensembles

**Fig 5:**
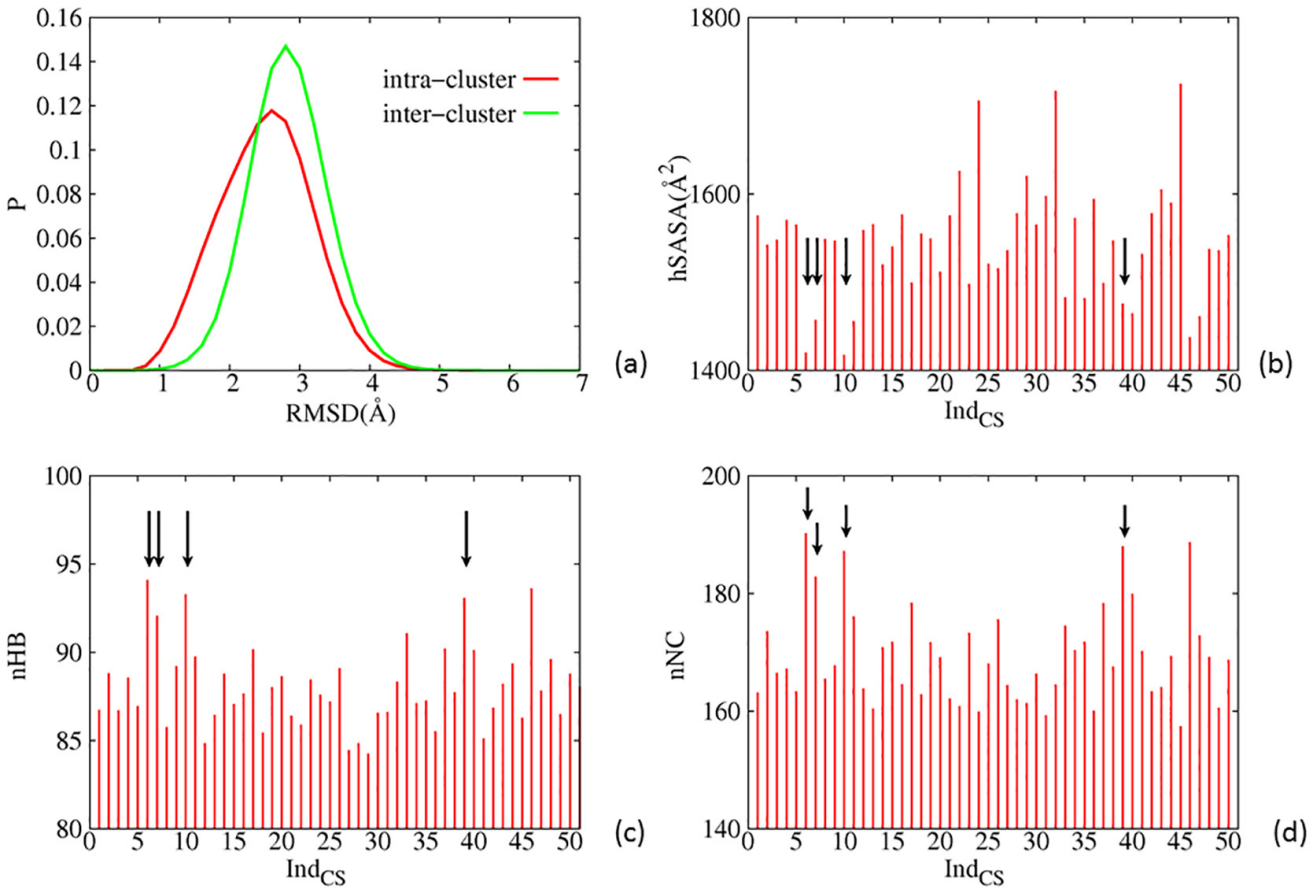
A few physical properties of major clusters at temporal resolution *T*_4_, a) Distributions of intra-cluster (red) and inter-cluster (green) pwRMSD. b) *hSASA*, c) *nHB* and d) *nNC* of the 50 largest clusters. Arrows in b), c) and d) indicate clusters harboring crystal structures.

To examine structural differences within each and among different clusters. We calculated the pair-wise root mean squared deviation (pwRMSD) based on backbone atoms, and the results are shown in Fig 5a. At temporal resolution *T*_4_, inter-cluster structural differences as measured by pwRMSD are on average slightly larger than that within each cluster, as one would intuitively expect. However, distributions for inter-cluster and intra-cluster pwRMSD are both single-peaked and largely overlap. A few other molecular scale physical quantities, including the number of hydrogen bonds (*nHB*), the number of native contacts (*nNC*), radius of gyration (*R*_*g*_), and *hSASA* were calculated for major CSs at multiple temporal resolutions. As shown in Fig 5(b)–5(d) and Fig. F in S1 File, CSs occupied by crystal structures exhibit significantly larger *nHB*, *nNC* and smaller *hSASA* than other ones, while sharing similar average *R*_*g*_.

**Fig 6:**
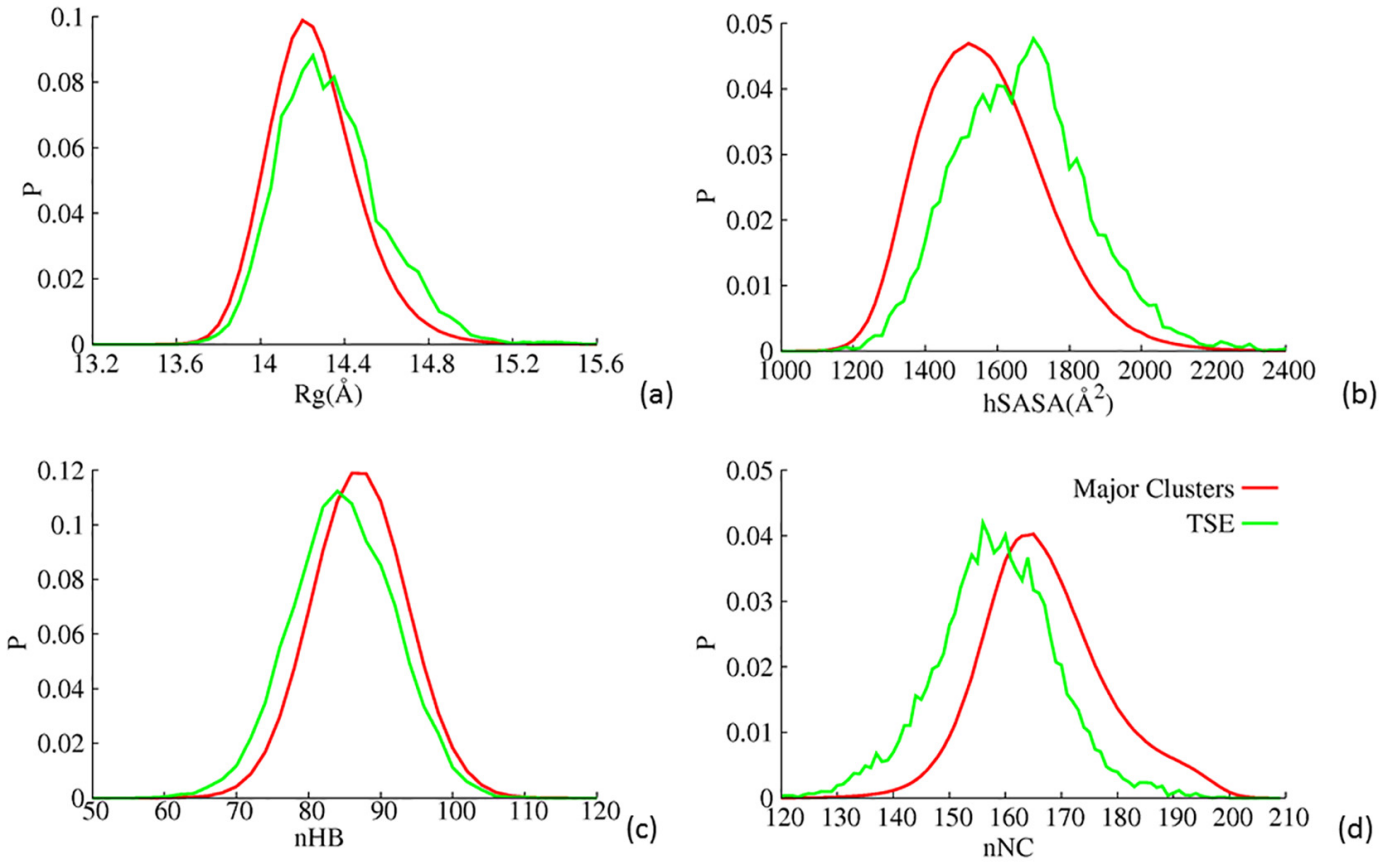
Distributions of a)*R*_*g*_, b)*hSASA*, c)*nHB* and d)*nNC* for major clusters (the 50 largest ones) and TSEs at temporal resolution *T*_2_.

We also calculated these quantities for transition state ensembles (between CSs). As shown by Fig 6, At temporal resolution *T*_2_, transition state ensembles at various temporal resolutions exhibits larger *hSASA*, larger *R*_*g*_, smaller *nHB* and *nNC*. Similar but less significant observations were made at other temporal resolutions (see Fig. G in S1 File). These observations suggest that transition structural states corresponding to change of backbone torsional states are more disordered and expanded. Disordering within native state of well-structured protein is not an established concept. Due to their small statistical significance in equilibrium conformational distributions, these transition state ensembles have trivial impact on thermodynamic properties. For the same reason, direct experimental comparison is extremely difficult at present. However, large-amplitude backbone dihedral rotations are important in protein conformational dynamics. Therefore, further investigation in this aspect is necessary.

### Life time of transition state ensembles at multiple temporal resolutions

**Fig 7:**
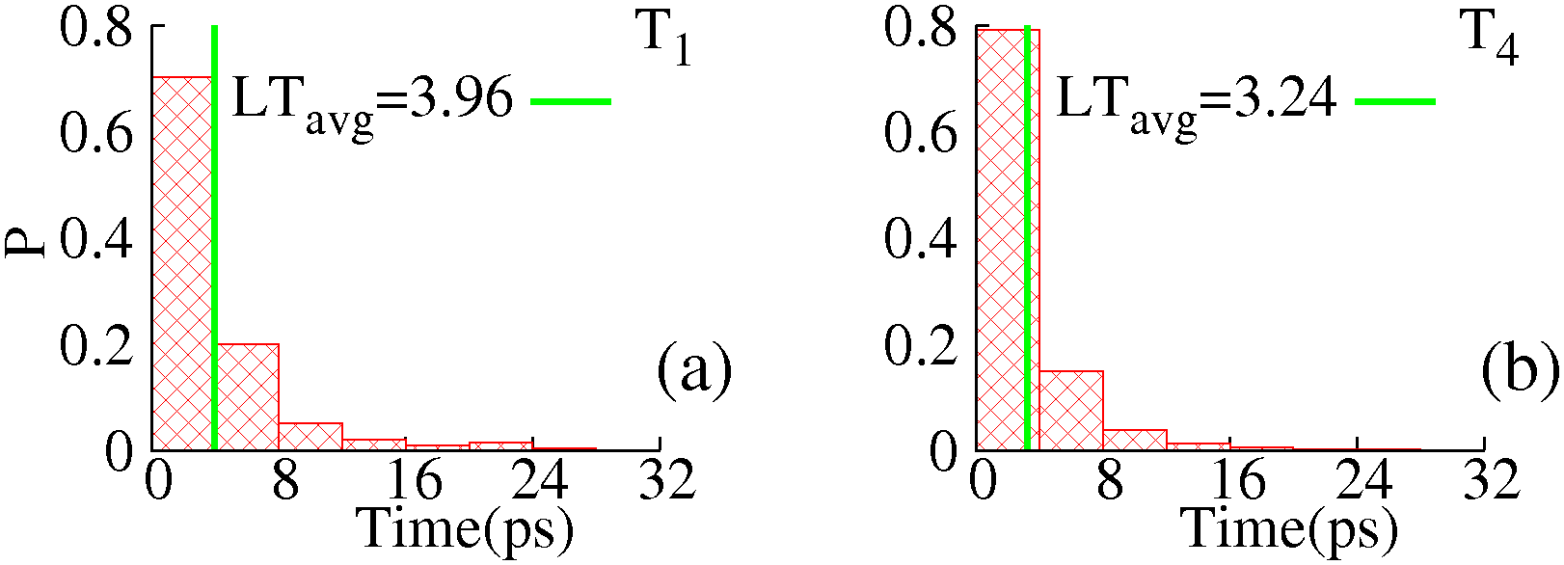
Distributions of TSE life times at temporal resolution a) *T*_1_ and b) *T*_4_. The green impulses indicate estimated averages (*LT*_*avg*_).

Life time for TSEs of protein folding have been investigated extensively by experimental, theoretical and computational studies and found to vary from multiple *ps* to hundreds of *ns* [31–34]. No quantitative characterization of life time for TSE of backbone torsional transitions in native protein is available to the best of our knowledge, however. We calculated averages and distributions of life times for TSEs at various temporal resolutions and plotted the results in Fig 7. It is interesting that both distributions and averages of torsional transition times are similar for all backbone torsions that were used in clustering, regardless of the average waiting times, which are used to define time scales for corresponding torsional DOFs and range over five orders of magnitude. As cluster splitting backbone torsions at various temporal resolutions are distributed across the whole primary sequence (Table A in S1 File), this observation implies that life time of backbone torsional transition state is insensitive to local environment. While specific life times for transition state of backbone torsional DOFs might vary for different force fields, its observed invariance across primary sequences implies insensitivity to local environment, which is likely to be a general feature of proteins.

### Side chain torsional degrees of freedom

Side chain torsional DOFs, together with backbone ones, are generally termed “soft” DOFs and have been demonstrated to be the major source of protein configurational entropy [35]. We calculated distributions of *χ*_1_ for all rotatable side chains that have at least one heavy atom beyond *C*_*β*_. It is found that each *χ*_1_ significantly populate all three major rotameric states (Fig. H in S1 File). Waiting time for these interconvertions ranging from a few nanoseconds to microseconds and beyond (See Fig. I in S1 File). Addition of *χ*_1_s in the clustering results in a dramatic increase of cluster number at each temporal resolution, and *N*_*CS*_ amounts to 7382009 at temporal resolution *T*_4_. Further addition of *χ*_2_ increases *N*_*CS*_ to 38437034 at *T*_4_. These observations reflect significantly less correlations among side chains when compared with backbone dihedrals as calculated by us (Fig. J in S1 File) and reported elsewhere [36], and confirm the conclusion from an earlier study [37] that globular proteins have solid like backbone DOFs and liquid like side chain DOFs. These side chain based clusters are not helpful in understanding experimental observables and function of proteins since each single cluster has negligible statistical weight. Therefore, we did not perform further analysis on them. This observation by no means negates the importance of side chain flipping in function of some proteins (e.g. open/close of a channel) [38, 39], where it is the marginal probability of a specific one or a few side chain torsional state(s), not the probability of a unique side chain torsional state combination (which is always negligible in statistical weight), that accounts for the corresponding structural, dynamical or functional importance.

## Discussion

### Selection of clustering criteria and methodology

In folding and design studies, RMSD from a specific native structure (usually a crystal structure) or pwRMSD is widely utilized for clustering and a number of accelerated methods are developed for this purpose [40–42]. This is quite effective as structural differences between native ensemble and various unfolded ensembles are significant. Another type of widely used quantities are principal components (PC) obtained from diagonalizing the covariance matrix [43]. Principal component analysis (PCA) has been shown to be effective in reducing dimension of protein FEL as first few PCs usually dominate the whole FEL that is explored by simulations on the order of hundreds of nanoseconds [16]. This is not necessarily true for very large number of snapshots exhibiting strong structural diversity. When PCA is applied to snapshots from single or a few 100−*ns* HEWL trajectories, in agreement with many previous studies, the first few PCs dominate the whole FEL. However, when snapshots from hundreds of 100−*ns* trajectories were used, strong structural diversity renders the first few PCs much less dominant (Fig. K in S1 File). It is important to note that PCA analysis is based on the structural covariance matrix, which does not have any time scale related information. The dominating eigenvectors corresponding to collective motions that are of the largest spatial magnitude, not necessarily the longest time scale. Therefore, both RMSD and PCs (from PCA) are not in line with our major goal of analyzing the explored conformational space at multiple temporal resolutions.

Markov state models (MSM) is a powerful technique with explicit temporal resolution consideration, and has been successfully applied to analyze major functionally important metastable states for many proteins [44]. There have been significant improvement of kinetic network models, such as the super-level-set hierarchical clustering [45] and Hierarchical Nyström methods [46] for multiple resolution analysis of bimolecular dynamics, since earlier days of MSM [47]. We initially chose to use distributions of backbone dihedrals for clustering based on the well-acknowledged importance of these torsional DOFs for protein conformational transitions, the deterministic clustering results, and explicit consideration of temporal resolution. When combined with the radix sort algorithm [48] and bit-encoded torsional states, the clustering process may be accomplished with trivial computational cost. The above discussed results indicate that clustering based on backbone torsional states divide crystal structure ensemble into physically meaningful sub-clusters. Additionally, each backbone torsional DOF is associated with a specific residue, potentially rendering easy integration of conformational analysis with sequence analysis and mutation experiments. Explicit knowledge of time scales and distributions of individual backbone torsions in specific environment may be utilized by machine learning methods for prediction of CS distribution and dynamics. Therefore, given its low computational cost, conceptional simplicity and practical utility, backbone dihedral distribution based clustering is a useful approach for conformational analysis of proteins. Apparently, MSM is a more theoretically advanced methods for multiple-resolution analysis of MD trajectory sets. However, specifying number of clusters for different hierarchies, which we do not have much information *a priori*, becomes a challenge. Additionally, the clustering itself become computationally intensive for large number of snapshots as in our case. It would be interesting to have a detailed comparison between our backbone dihedral based clustering and MSM analysis in the future.

### The significance of MD simulation results and the crystal structural ensemble

While admitting that each current force field parameter set has its own limitations, it is certainly true that they describe very realistic models of proteins. The significance of simple lattice model [49–51] and Go model [52, 53] studies can not be over emphasized in developing protein folding theories. Recent atomistic simulations of the folding of many small globular proteins [24], while greatly enriched original simple models, proved rather than negated the significance of their major conclusions. At microsecond temporal resolution (*T*_2_), dozens of transitions are observed between the dominating cluster that harboring nearly all crystal structures and other small clusters, suggesting that the force fields is effective in locating the major native structural states. Therefore we believe that our results, despite the fact that it does not strictly reflecting the global FEL of HEWL under physiological conditions, give a highly realistic model, and the conclusions regarding the topology and organization of CS are likely to be representative for many globular proteins.

Crystal structures of proteins, despite their being obtained from non-physiological conditions, have beautifully rationalized numerous critical molecular biological processes (e.g. central dogma related processes, energy production, signal transduction). This is consistent with our remarkable observation that 114 out of 120 crystal structures, which were obtained under widely different conditions, are located within a central 2 *μ*s bottom region of the native HEWL FEL explored by 0.2ms MD simulations. Five out of the remaining six crystal structures are obtained under extreme conditions such as low hydration and high sodium nitrate concentration (see supporting information for details). Our simulation results, together with the large crystal structural ensemble, suggest that HEWL is likely to have only one narrow major native FEL well (within a few microseconds) that harbors structural states to bind substrates and interact with other proteins. Since force fields are always defective to some extent, very long trajectories might get lost in unrealistic conformational states, running multiple moderately long trajectories in the vicinity of high resolution experimental structures may alleviate this artifact to some degree. Therefore, to sample conformational space of proteins with similar type of native FEL in the vicinity of a crystal structure, utilizing multiple independent microseconds-long MD trajectories might be a better strategy than running a single milli-second or longer MD trajectory.

### Conclusions and the prospects

In this study we performed a hierarchical conformational analysis for HEWL, a typical globular protein with sufficient structural complexity, at multiple temporal resolutions ranging from *ns* to *μs*. We observed hub-like topology at microsecond and coarser temporal resolutions, and increasingly diversified structural states and more connectivity among CS at increasingly finer temporal resolutions. Various molecular interactions captured in CSE were found to associate with CS transitions covering time scales from nanoseconds to microseconds. CSE for HEWL are found to be associated with kinetic-trap clusters at ∼ 10–100*ns* temporal resolution. The number of CS that have non-negligible statistical weight is quite limited, even at *ns* temporal resolution. These observations suggest that to study CS of native globular proteins at temporal resolutions ranging from *ns* to *μs* is both important and potentially feasible for prediction of molecular interactions. However, our study is limited to one specific protein. The apparent immediate questions needs to be answered are: i)How is distribution and organization of CS related to specific protein size, folds and sequences; ii)What are the roles of hierarchical CS in protein-protein and protein-ligand interactions. We are working on a few more proteins with different sizes and folds to further increase our understanding in these directions. we hope that this work will stimulate more investigations in this line.

## Methods

### Molecular dynamics simulations

MD simulations systems were setup and equilibrated with VMD [54] and NAMD [55]. Production runs were performed using ACEMD [56]. 120 crystal structures of HEWL, including 44 in protein-protein-interaction complexes and 76 in free form (or with bound small molecules), were taken from PDB (www.pdb.org). Each structure is solvated with 6575 TIP3P water molecules and neutralized with 5 *Na* ^+^ and 13 *Cl* ^−^ ions, resulting in a system with 21703 atoms. The 120 HEWL simulation systems were first equilibrated for 200ps in NVT ensemble and for 1ns in NPT ensemble, both harmonic restraint on protein heavy atoms. After that, restraints were released and 3ns of NPT run were performed to obtain a proper volume (a cubic box with 59.582 *Å* sides) that is used in the following NVT production runs. Starting configurations for production NVT runs were selected from the last 1ns of the previous NPT run with the criteria of having right box size (within 0.01*Å*). CHARMM22 (with CMAP) force fields were utilized with a 9*Å* non-bonded cutoff distance in production runs. The simulation time step is 2*fs* for equilibration with NAMD and 4*fs* with hydrogen repartition for production run with ACEMD. Electrostatics were treated by PME with gird size being approximately 1*Å*. 20 independent trajectories were started from each equilibrated PDB structure by using 20 different random number seeds for initial atomic velocity assignment, resulting in 2400 initial trajectories. Trajectories stopped due to machine failure (mainly writing error due to full disks, GPU memory error and operating system failing) were discarded. 2000 100 − *ns* trajectories, with collectively 50,000,000 snapshots, were utilized for final analysis.

### Clustering of structural and transition state ensembles at various temporal resolutions

Backbone dihedral distributions and torsional states. Distribution of each backbone dihedral angle (*ϕ*, *ψ*) is constructed by using histograms with bin size being 1°. To perform clustering, local minima are found for each backbone dihedral distribution. Whenever the angle distance between two local minima of a given dihedral is smaller than 60, the minimum with smaller probability is taken as an effective. After this initial filtering, a backbone dihedral is selected as a potential clustering dihedral if two or more effective local minima exist. Each region between two neighboring local minima is defined as a torsional state for that torsional DOF. As dihedral angles are cyclic variables, the number of state is equal to the number of local minima utilized for splitting corresponding dihedrals. We found that 127 backbone dihedrals (out of the total of 256) exhibit two-peak distributions and are defined as two-state torsional DOFs that were used in clustering.

Time scales. The time scale for each specific two-state backbone dihedral is defined by the average waiting time between two transition events regardless of its specific directions and routes (For circular two-state system with state *A*, *B* and minima *MIN*_*A*_, *MIN*_*B*_, there are two possible directions and four possible routes, *A* → *MIN*_*A*_ → *B*, *A* → *MIN*_*B*_ → *B*, *B* → *MIN*_*A*_ → *A*, *B* → *MIN*_*B*_ → *A*,). Specifically, within our collective trajectory time of 200*μs*, if *N* transitions (*N*_*trans*_) happened between the two states of an given dihedral, then the average waiting time is $t_{w}=\frac{200}{N_{trans}}\mu s$, which is used to define time scale of the corresponding torsional DOF.

Clustering at five temporal resolutions. To establish backbone based hierarchical clustering, we first divided all 127 two-state backbone dihedrals into five groups corresponding to five different temporal resolutions *T*_0_ (20*μs* < *t*_*w*_ < 200*μs*), *T*_1_ (2*μs* < *t*_*w*_ < 20*μs*), *T*_2_ (200*ns* < *t*_*w*_ < 2*μs*), *T*_3_ (20*ns* < *t*_*w*_ < 200*ns*) and *T*_4_ (2*ns* < *t*_*w*_ < 20*ns*). Two structures are assigned to the same cluster only if for each clustering participating torsional DOF, both structures are in the same torsional state. When performing clustering at a given temporal resolution, two-state backbone dihedrals with shorter time scales are treated as single-state DOF and are excluded. For example, when clustering at temporal resolution *T*_2_, we use backbone dihedrals that have time scales within the range of *T*_0_, *T*_1_ and *T*_2_, but ignore those with time scales within the range of *T*_3_ and *T*_4_. Due to limited statistics, we did not perform detailed analysis at temporal resolution *T*_0_. Backbone torsional DOFs utilized for each temporal resolution is listed in Table A in S1 File.

Transition state ensembles. First we define transition state ensemble for effective local minimum of all two-state backbone torsional DOFs (*ϕ* and *ψ*) as follows. At temporal resolutions *T*_3_ and *T*_4_, a 5 degree region (2.5 degree to each side of an effective local minimum) is defined as the transition state. At temporal resolutions *T*_0_, *T*_1_ and *T*_2_, for a given local minimum of a specific torsional distribution, number of snapshots in 2.5 degree bins to each side of the minimum are assigned to *N*_+1_, *N*_+2_, *N*_+3_, ⋯ and *N*_−1_, *N*_−2_, *N*_−3_, ⋯. If *N*_±(*i*+2)_ − *N*_±(*i*+1)_ ≫ *N*_±(*i*+1)_ − *N*_±*i*_,(*i* = 1, 2, 3, ⋯), then bins 1, ⋯, *i* + 1 are defined as the transition state region of the corresponding local minimum. Due to finite sampling, limited connectivity on each level of hierarchical conformational space, and correlations between (among) different backbone torsional DOF, local minima in distributions of each two-state torsional DOF do not necessarily correspond to the transition ensembles between different clusters. Only when transition state of a specific DOF coincide with transition between two clusters at a given temporal resolution, it is taken as transition state of corresponding clusters and snapshots within that state are counted as transition state structures.

Principal component analysis. Dihedral principal component analysis (dPCA) is utilized in this study. To tackle the circular property of dihedral angles, we used the following trigonometric functions to represent backbone dihedrals (*ϕ* and *ψ*) [57]. *q*_2*i*−1_ = cos *ϕ*_*i*_, *q*_2*i*_ = sin *ψ*_*i*_, with *i* running through the number of residues. Subsequently, covariance matrices *σ*_*ij*_ = ⟨(*q*_*i*_ − ⟨*q*_*i*_⟩)(*q*_*j*_ − ⟨*q*_*j*_⟩)⟩ are constructed for selected (or all) trajectories and diagonalized to obtain principal components. We also performed PCA analysis based on cartesian coordinates for protein backbone atoms.

### Calculation of various physical properties

Radius of gyration. Radius of gyration for each snapshot is calculated as $r_{g}=\sqrt{\frac{\sum_{i=1}^{n_{atom}}m_{i}r_{i}^{2}}{\sum_{i=1}^{n_{atom}}m_{i}}}$, with *m*_*i*_ and *r*_*i*_ being the mass and the distance to the molecular center of mass for atom *i* respectively, *n*_*atom*_ is the total number of atoms in HEWL.

Native contacts. A residue contact is defined for two non-sequential residues with *C*_*α*_ distance being smaller than 6.5*Å*. Residue contacts that are shared by two thirds (80) or more of the 120 crystal structures utilized in this study are defined as native contacts.

Hydropobic solvent accessible surface area (hSASA). A 1.4*Å*-diameter sphere is used to probe HEWL surface of hydrophobic residues (as defined by VMD) with VMD [54].

Hydrogen bonds. Existence of a hydrogen bond is defined by a distance between a donor and an acceptor being smaller than 3.5*Å* (cutoff distances ranging from 3.0*Å* to 3.5*Å* gives similar ordering among different clusters when the number of hydrogen bonds is compared), and by the corresponding “donor → H → acceptor” bend angle being larger than 130.

## Supporting Information

S1 File (PDF)
