# Supplementary material for: Hierarchical Conformational Analysis of Native Lysozyme Based on Sub-Millisecond Molecular Dynamics Simulations
Source: PLoS One. 2015 Jun 9;10(6):e0129846. doi: 10.1371/journal.pone.0129846 (PMC4461368; doi:10.1371/journal.pone.0129846)
Supplement: S1 File — (PDF) [file pone.0129846.s001.pdf]

Hierarchical conformational analysis of native lysozyme based on sub-millisecond molecular dynamics simulations

Kai Wang[1], Shiyang Long[1], and Pu Tian[1,2]

[1]College of Life Science
[2]Key Laboratory of Molecular Enzymology and Engineering of the Ministry of Education , Jilin University, 2699 Qianjin Street, Changchun China 130012

May 21, 2015

Supporting information

Text A Clustering of HEWL crystal structure ensemble and relevant molecular interactions

For short, PDB codes are used to represent each crystal structure below, and clusters at various temporal resolutions are named as CS-n-T_m, representing the nth largest cluster at temporal resolution T_m. At temporal resolution T_0, all crystal structures are located in the dominating cluster that have an overwhelming statistical weight of 99.962%.

At temporal resolution T_1, CS-1-T_0 (the dominating cluster on T_0) splits into 22 clusters, with 118 crystal structures in CS-1-T_1. One additional CS, CS-2-T_1, harbors 2 crystal structures 1XEK and 1KIP, which have a different torsional state of ϕ128 when compared with CS-1-T_1. 1XEK is crystallized under a low hydration rate of 9.4%. 1KIP is in complex with a mutated antibody FvD1.3

that is designed to eliminate inter-molecular hydrogen bonds between the wild type antibody and HEWL.

At temporal resolution T_2, CS-1-T_1 split into 282 clusters, within which CS-1-T_2 is the largest cluster and harbors 114 crystal structures. The other crystal-structure-harboring cluster is CS-5-T_2 that has $1XEI, 1XEJ, 2FBB$ and $1ZVY$. CS-1-T_2 and CS-5-T_2 have distinct torsional state for $\phi 128$. $1XEI$ and $1XEJ$ are crystallized under low hydration levels of 17.6% and 16.9% respectively. $2FBB$ is obtained under high sodium nitrate concentration, with nitrate ions form a ring-like distribution around the HEWL in the crystal unit cell. $1ZVY$ has a phosphate group directly interact with $R128$ of HEWL.

At temporal resolution T_3, CS-1-T_2 splits into 3930 clusters, five of which harboring crystal structures are CS-5-T_3, CS-6-T_3, CS-10-T_3, CS-828-T_3 and CS-6775-T_3, with corresponding number of crystal structures being $63, 4, 45, 1$ and 1 respectively. CS-5-T_3 and CS-10-T_3 have different torsional states for $\phi 74$. $1HEW$, a complex of HEWL and its inhibitor NAG_3, belongs to CS-5-T_3, while HEWL structures from complexes with other reactive sugar substrates ($1SF4, 1SF5, 1SF6, 1SFB, 1SFG$) belong to CS-10-T_3. Although $N74$ is far from the active cleft region between $E35$ and $D52$, the strain caused by substrate binding in this region is reflected at distal $N74$. While the detailed mechanism of coordinated allostery is beyond the scope of this study, the time scale of relevant conformational change in active region should be commensurate with that observed for $\phi 74$. Four crystal structures in CS-6-T_3 are from complexes with various mutant antibody HyHEL-10 Fv ($3A67 : LN31D$, $1J1O : LS91A$, $1J1X : LS93A$ and $2DQG : HY53F$), while HEWL in complex with wild type HyHEL-10 Fv and a few other mutant HyHEL-10 Fv are in CS-5-T_3. Residues $LS91$ and $LS93$ of the light chain of HyHEL-10 Fv are in close approximity with $N19$ of HEWL and corresponding mutations eliminated hydrogen bonds

at the HEWL-antibody interface. $LN31$ and $HY53$ from light and heavy chain of HyHEL-10 Fv is far from $N19$ of HEWL but resulted in the same change of torsional state for DOF $\phi19$. CS-828-T_3, separated from CS-5-T_3 by $\phi118$, harbors a HEWL crystal structure extracted from $1MLC$, a complex between HEWL and antibody $D44.1$, which has a 1000 fold lower affinity with HEWL than HyHEL-10 Fv. CS-6775-T_3, separated from CS-5-T_3 by $\psi25$, is obtained from a very low temperature (124K) with multi-wavelength anomalous solvent contrast (MASC) data.

At temporal resolution $T4$, CS-5-T_3 splits into 64 clusters, two of which harboring crystal structures are CS-6-T_4 and CS-39-T_4, with 55 and 8 crystal structures respectively. CS-39-T_4 is separated from CS-6-$T4$ by the torsional DOF $\psi85$, $\phi18$ and $\phi85$, the 8 crystal structures within which are from complexes with the monoclonal antibody D1.3 and its various point mutants.

	$N_{torsion}$	Lists of torsional DOFs
T_0	9	φ_5, φ_{10}, φ_{14}, φ_{54}, φ_{58}, φ_{61}, φ_{77}, φ_{82}, φ_{87}
T_1	16	ψ_5, φ_{13}, φ_{38}, φ_{39}, φ_{40}, φ_{45}, φ_{51}, ψ_{58}, ψ_{61}, φ_{62}, ψ_{63}, φ_{63}, ψ_{69}, φ_{112}, φ_{113}, φ_{128}
T_2	29	φ_1, φ_2, ψ_7, ψ_{10}, ψ_{11}, ψ_{12}, ψ_{13}, φ_{16}, ψ_{32}, φ_{34}, φ_{35}, φ_{37}, φ_{41}, φ_{43}, φ_{44}, ψ_{53}, φ_{53}, φ_{64}, φ_{65}, φ_{67}, φ_{75}, φ_{76}, ψ_{81}, ψ_{95}, ψ_{98}, φ_{114}, φ_{122}, φ_{123}, φ_{127}
T_3	53	ψ_2, ψ_4, ψ_{14}, φ_{17}, φ_{18}, φ_{19}, φ_{20}, φ_{22}, φ_{23}, ψ_{25}, φ_{25}, ψ_{26}, ψ_{33}, φ_{33}, ψ_{34}, ψ_{35}, φ_{36}, ψ_{37}, ψ_{39}, ψ_{40}, φ_{42}, ψ_{43}, φ_{46}, φ_{47}, φ_{49}, φ_{50}, ψ_{51}, φ_{56}, φ_{71}, φ_{72}, φ_{73}, φ_{74}, φ_{80}, φ_{84}, φ_{85}, φ_{99}, φ_{100}, φ_{102}, φ_{104}, φ_{106}, φ_{107}, ψ_{109}, φ_{109}, ψ_{111}, φ_{111}, ψ_{112}, ψ_{114}, φ_{117}, φ_{118}, φ_{119}, φ_{120}, φ_{124}, φ_{126}
T_4	20	ψ_{36}, ψ_{44}, ψ_{56}, ψ_{60}, φ_{60}, ψ_{64}, ψ_{65}, φ_{68}, ψ_{75}, ψ_{76}, ψ_{85}, φ_{86}, ψ_{99}, φ_{105}, ψ_{108}, ψ_{110}, ψ_{113}, φ_{115}, ψ_{120}, ψ_{123}

Table A Number ($N_{torsion}$) and lists of backbone torsional DOF that were utilized for clustering for each defined temporal resolution.

Timescale	Distribution of crystal structures
T_1	118 in $CS-1$, 1XEK,1KIP in $CS-2$
T_2	114 in $CS-1$, 1XEI,1XEJ,2FBB,1ZVY in $C-5$<br>1KIP in $CS-11$, 1XEK in $CS-234$
T_3	63 in $CS-5$ (1SFB, 1UC0, 1YL1, 1WTM, 1SF7, 1WTN, 1HSW, 4LZT, 2ZQ3, 1HSX, 1AT<br>1LZN, 1F10, 1F0W, 1HEL, 1VDT, 1VED, 1B0D, 1IO5, 1SF6, 1YL0, 1VDQ, 1V7S, 1AKI, 2<br>3A6B, 2ZNW, 2DQC, 2DQD, 1C08, 1UA6, 1IC5, 1RI8, 2DQJ, 1KIR, 1B2K, 1G7M, 1T6V,<br>1G7I, 1YQV, 1G7J, 1ZV5, 1VFB, 1P2C, 1G7H, 1IC7, 2DQF, 3D9A, 2DQH, 1XGP, 1DQJ,<br>2XTH, 2LYZ, 1JIY, 2W1Y, 6LYZ, 1YKX, 2H9J, 1BWJ, 1PS5, 1YKZ, 2W1L, 2EPE, 1BVX<br>2W1X, 1Z55, 1W6Z, 1JIT, 1VDS, 3M3U, 1YKY, 2I6Z, 1BWI, 1GWD, 1YIK, 2ZQ4, 1ZMY,<br>2FBB in $CS-127$, 1KIP in $CS-150$, 1MLC in $CS-828$, 1BHZ in $CS-6775$<br>1XEI,1XEJ in $CS-7898$, 1XEK not find
T_4	55 in $CS-6$ (1SFB, 1UC0, 1YL1, 1WTM, 1SF7, 1WTN, 1HSW, 4LZT, 2ZQ3, 1HSX, 1AT<br>1RFP, 1LZN, 1F10, 1F0W, 1HEL, 1VDT, 1VED, 1B0D, 1IO5, 1SF6, 1YL0, 1VDQ, 1V7S, 1<br>2DQC, 2DQD, 1C08, 1UA6, 1IC5, 1RI8, 2DQJ, 1B2K, 1T6V, 3A6C, 2DQE, 1IC4, 1YQV, 1<br>4 in $CS-7$ (3A67, 1J1O, 1J1X, 2DQG), 4 in $CS-7$ (3A67, 1J1O, 1J1X, 2DQG), 45 in C<br>2XTH, 2LYZ, 1JIY, 2W1Y, 6LYZ, 1YKX, 2H9J, 1BWJ, 1PS5, 1YKZ, 2W1L, 2EPE, 1BVX<br>2W1X, 1Z55, 1W6Z, 1JIT, 1VDS, 3M3U, 1YKY, 2I6Z, 1BWI, 1GWD, 1YIK, 2ZQ4, 1ZMY,<br>8 in $CS-39$ (1KIQ, 1KIR, 1G7M, 1G7L, 1G7I, 1G7J, 1VFB, 1G7H) ,1ZVY in $CS-63$, 2F<br>1MLC in $CS-1730$, 1XEI,1XEJ in $CS-22242$, 1BHZ and 1XEK not find

Table B Distributions of crystal structures among simulation derived conformational substates at defined temporal resolutions.

Mutant PDB code	Wild Type PDB code	Cluster number
W62Y(1LZD);W62Y(1LZE);W62F(1LZG)	1LZA;1LZB;1LZC	$CS-10-T_4$
N59D(3A3Q);N59D(3A3R)	1LZA	$CS-10-T_4$
S91T(1HEM);I55V,S91T(1HEN) I55V(1HEO);T40S,I55V,S91T(1HEP) T40S,S91T(1HEQ);T40S(1HER)	1HEL	$CS-6-T_4$
F3Y,H15L,Q41H,R73K V99A,D101G,Q121H(1UAC)	1UA6	$CS-6-T_4$
G49A(1FLQ);G67A(1FLU);G71A(1FLW) G102(1FLY);G117A(1FN5)		$CS-6-T_4$
D18A(1A2Y)	1G7L	$CS-39-T_4$
M12F,L56F(1IOQ);M12L,L56F(1IOR) M12F(1IOS);M12L(1IOT)		$CS-6-T_4$
I78M(1IR7);I58M(1IR8);I98M(1IR9)		$CS-6-T_4$
R68K(2IFF)		$CS-6-T_4$
D52N(3OJP);E35A(3OK0)		$CS-10-T_4$

Table C Distribution of 29 mutant structures among simulation derived conformational substates at temporal resolution T_4.

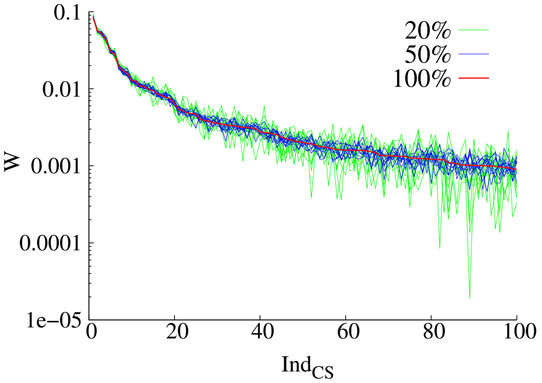

Figure A. Statistical weight (W) for the 100 largest clusters at temporal resolution T_4 obtained from all trajectories (red line). W for the same 100 clusters when calculated from subsets of trajectories that amount to 20% of the whole trajectories(green lines), and from subsets of trajectories that amount to 50% of the whole trajectories (blue lines).

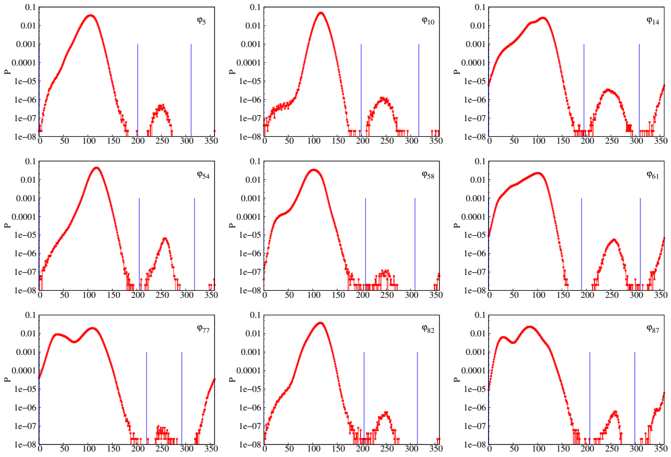

Figure Ba. Distributions of ψ and ϕ with waiting time t_w ranging from $20\mu s$ to 200 μs. Vertical blue lines indicate boundaries defining torsional states of the corresponding torsional DOFs.

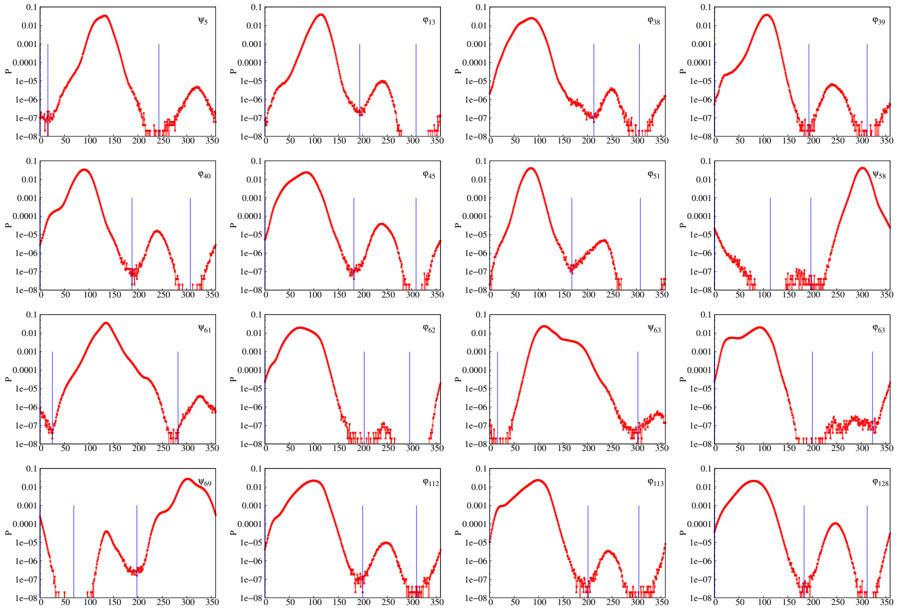

Figure Bb. Distributions of ψ and ϕ with waiting time t_w ranging from $2\mu s$ to 20 μs.

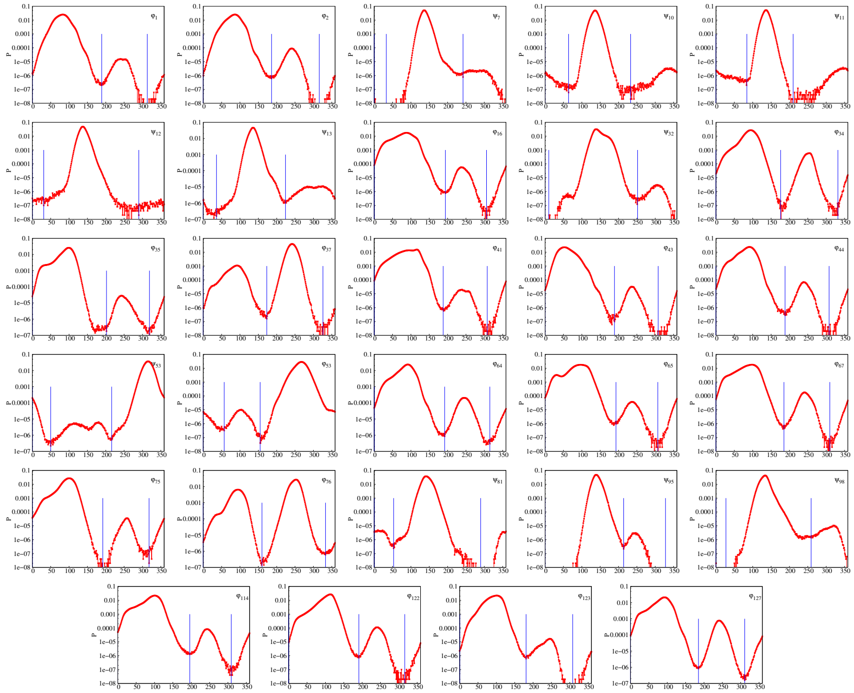

Figure Bc. Distributions of ψ and ϕ with waiting time t_w ranging from $200ns$ to $2\mu s$.

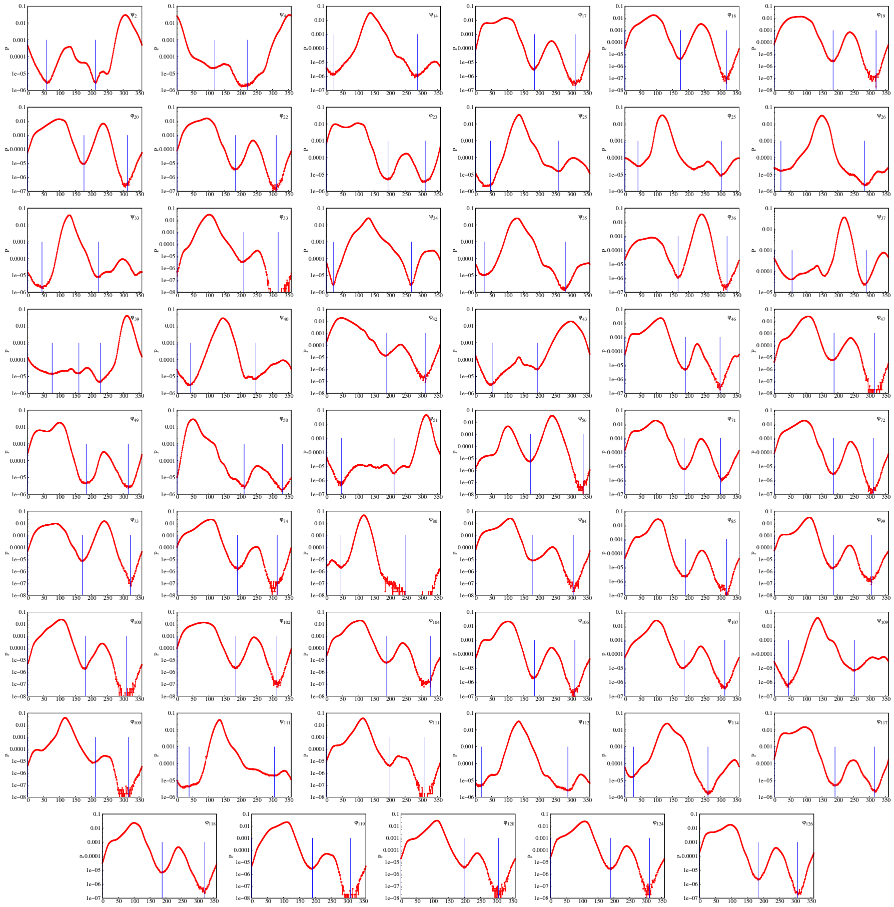

Figure Bd. Distributions of ψ and ϕ with waiting time t_w ranging from $20ns$ to $200ns$.

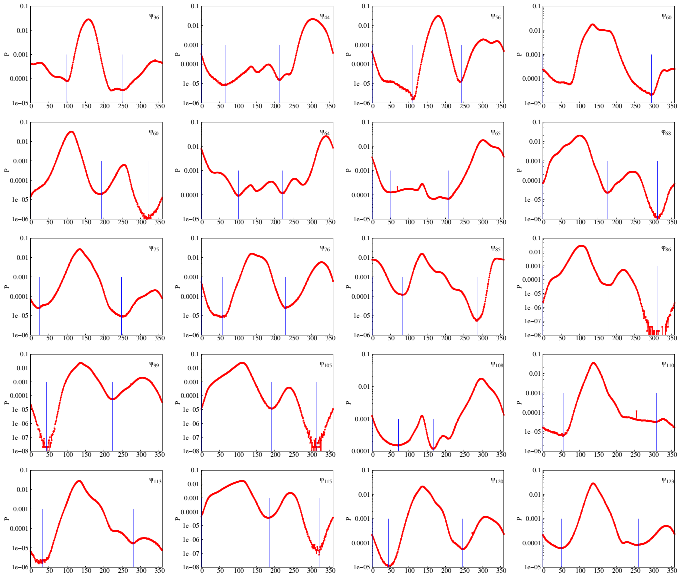

Figure Be. Distributions of ψ and ϕ with waiting time t_w ranging from $2ns$ to $20ns$.

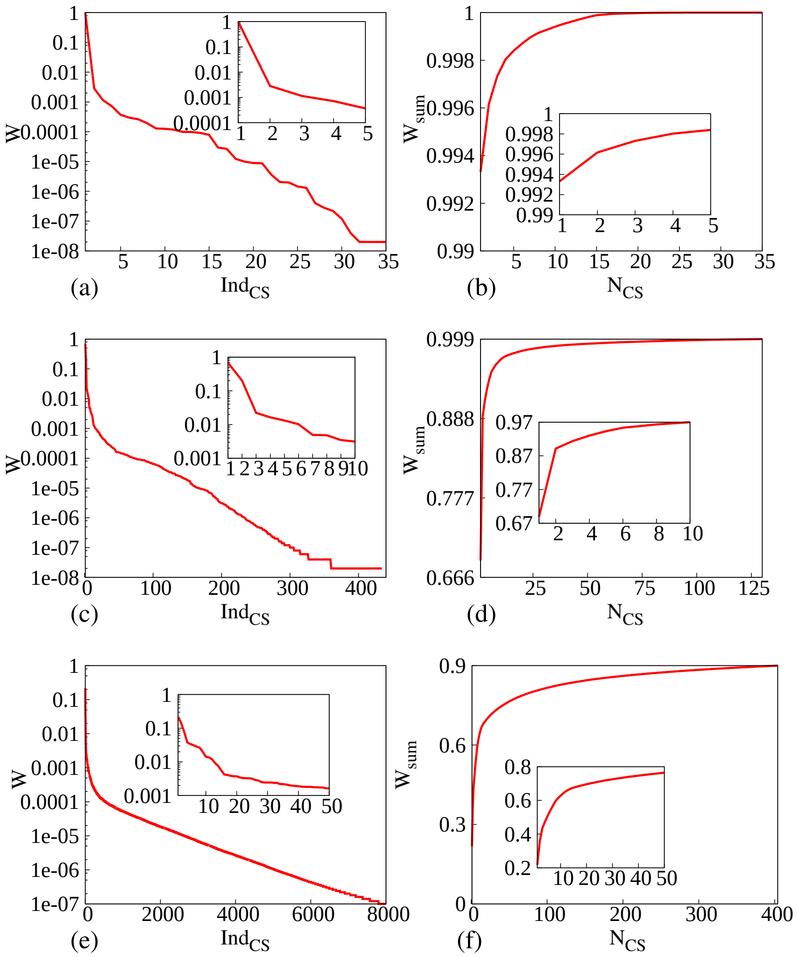

Figure C. Statistical weight (W) of the most significant CSs at temporal resolutions a) T_1, c) T_2 and e) T_3. horizontal axis is the index for CS (Ind_{CS}). Insets: magnification for small Ind_{CS}. Collective statistical weight (W_{sum}) of the N largest CSs (N_{CS}) at temporal resolutions b) T_1 , d) T_2 and f) T_3. Insets: magnification for small N_{CS}.

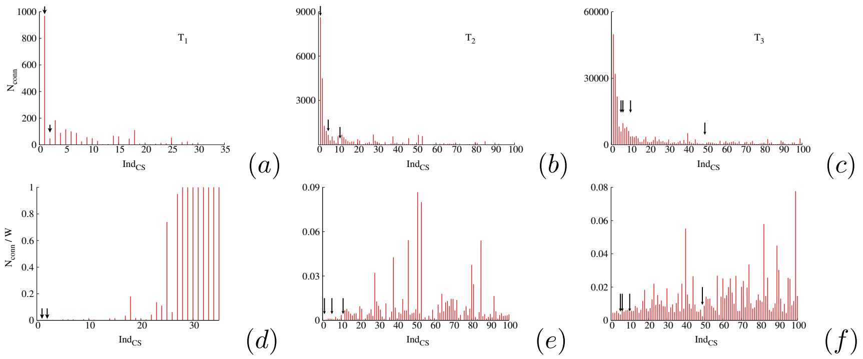

Figure D. Number of total inter-cluster transitions with (N_{conn}/W d,e,f) or without normalization (N_{conn} a,b,c) by statistical weights for each of the most significant CSs at temporal resolution T_1 (a,d), T_2 (b,e) and T_3 (c,f). CSs that harbor crystal structures are indicated by arrows.

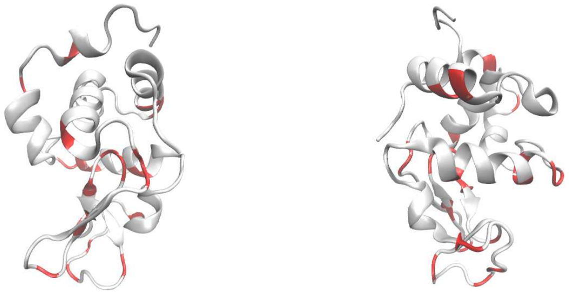

Figure E. Spatial distribution of point mutations in HEWL mutant crystal structure ensemble shown in two different views, red color indicate mutated residues.

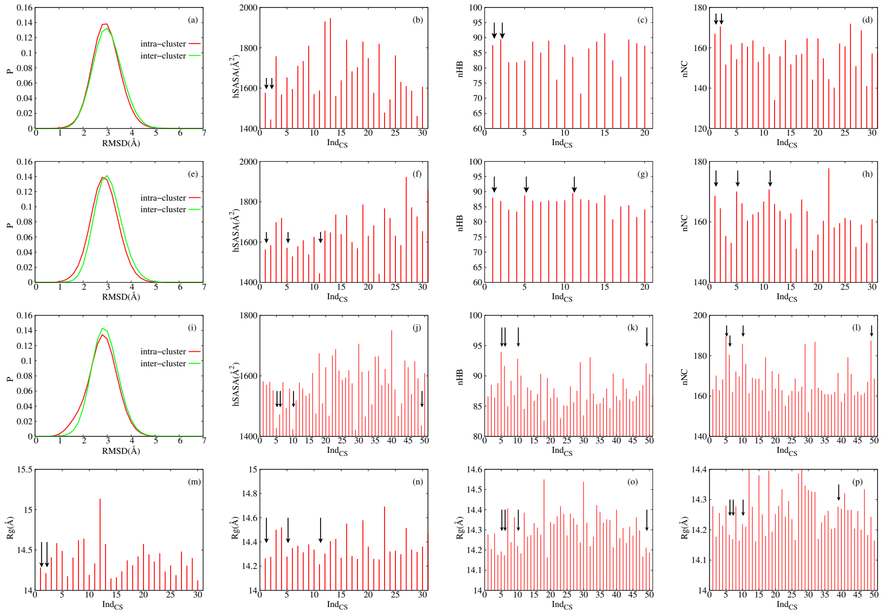

Figure F. The calculated physical properties for major clusters at temporal resolutions T_1, T_2, T_3. Distributions of intra- and inter-cluster pwRMSD, $hSASA$, nHB and nNC at temporal resolutions T_1 (a-d), T_2 (e-h) and T_3 (i-l). R_g for the major clusters are shown for temporal resolutions T_1 (m), T_2 (n), T_3 (o) and T_4 (p).

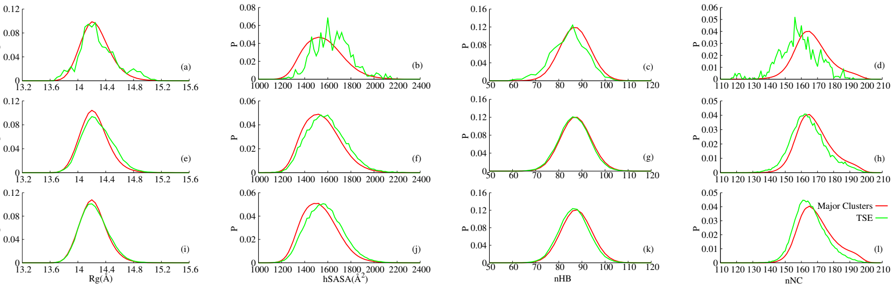

Figure G Distributions of R_g, $hSASA$, nHB and nNC for major clusters (the largest CS for T_1 and the 50 largest CSs for other temporal resolutions) and TSEs at temporal resolution T_1 (a-d), T_3 (e-h) and T_4 (i-l).

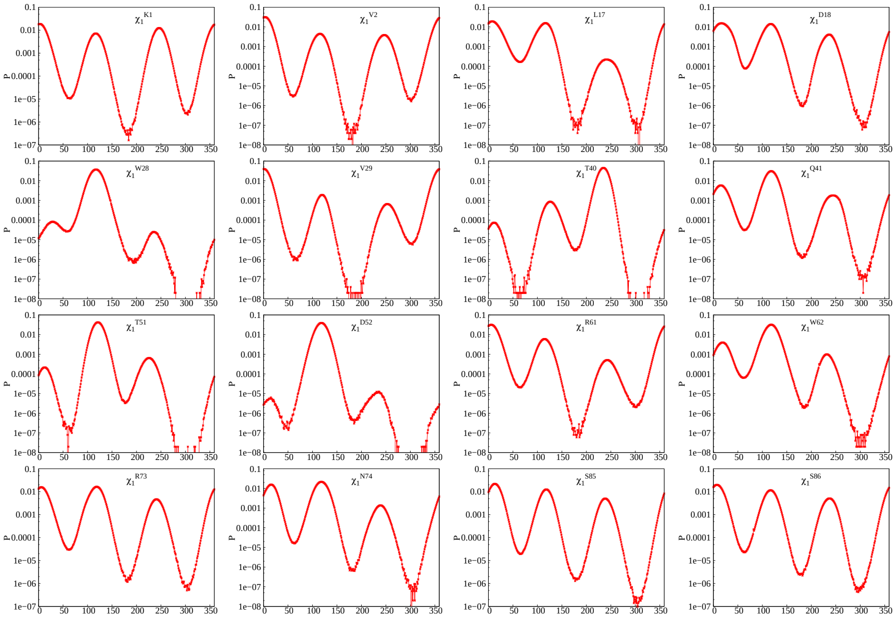

Figure H Distributions of 20 representative χ_1s. Superscripts are positions of corresponding residues in the primary sequence and their identity.

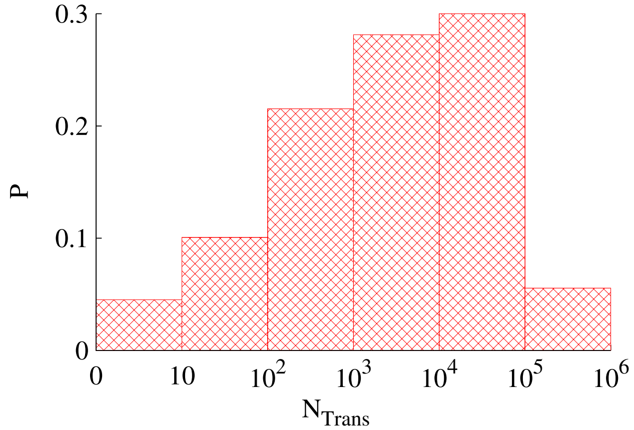

Figure I Distribution of the waiting time t_w for HEWL χ_1s obtained from our MD trajectories.

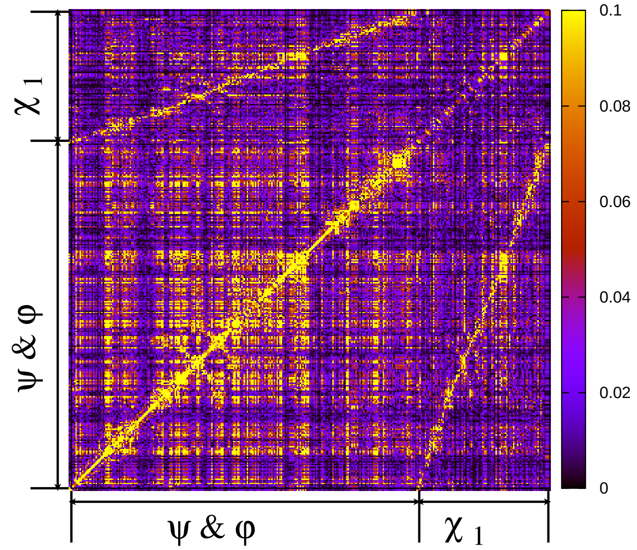

Figure J Observed pair correlations between all backbone torsional DOFs (ϕ and ψ) and heavy atom defined χ_1s.

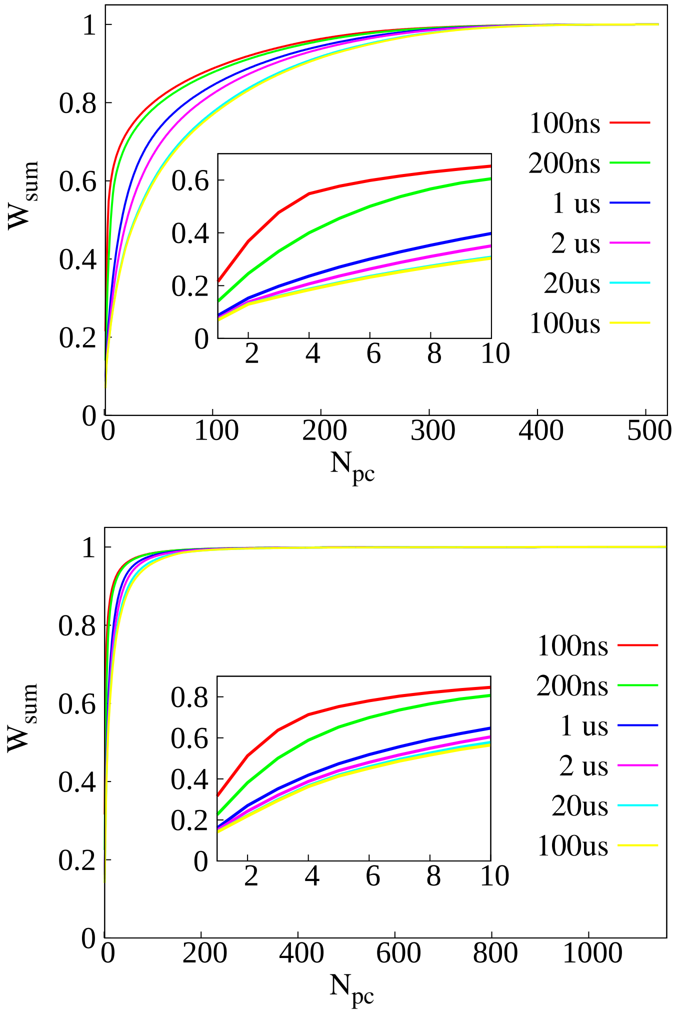

a)

b)

Figure K Accumulated weight of the first N principal components (N_{pc}) generated from various amount of trajectories. Data are averages from 100 selected single trajectories (for 100ns data) or 50 different combinations (other collective trajectory lengths). a)Results based on dPCA analysis, b)results based on PCA analysis with cartesian coordinates of protein backbone atoms.
